# Provider Costs of Treating Colorectal Cancer in Government Hospital of Malaysia

**DOI:** 10.21315/mjms2019.26.1.7

**Published:** 2019-02-28

**Authors:** Meram Azzani, Maznah Dahlui, Wan Zamaniah Wan Ishak, April Camilla Roslani, Tin Tin Su

**Affiliations:** 1 Community Medicine Department, Faculty of Medicine, MAHSA University, Saujana Putra Campus, 42610 Jenjarom, Selangor, Malaysia; 2 Centre for Population Health (CePH), Department of Social and Preventive Medicine, Faculty of Medicine, University of Malaya, 50603 Kuala Lumpur, Malaysia; 3 Department of Clinical Oncology, Faculty of Medicine, University of Malaya, 50603 Kuala Lumpur, Malaysia; 4 University of Malaya Cancer Research Institute (UMCRI), Faculty of Medicine, University of Malaya, 50603 Kuala Lumpur, Malaysia; 5 Department of Surgery, Faculty of Medicine, University of Malaya, 50603 Kuala Lumpur, Malaysia; 6 South East Asia Community Observatory (SEACO), Jeffrey Cheah School of Medicine and Health Sciences, Monash University Malaysia, 47500 Bandar Sunway, Selangor, Malaysia

**Keywords:** colorectal cancer, health care cost, Malaysia, provider perspective

## Abstract

**Background:**

The incidence of colorectal cancer (CRC) is rapidly rising in several Asian countries, including Malaysia, but there is little data on health care provider costs in this region. The aim of this study was to estimate the cost of CRC management from the perspective of the health care provider, based on standard operating procedures.

**Methods:**

A combination of top-down approach and activity-based costing was applied. The standard operating procedure (SOP) for CRC was developed for each stage according to national data and guidelines at the University of Malaya Medical Centre (UMMC). The unit cost was calculated and incorporated into the treatment pathway in order to obtain the total cost of managing a single CRC patient according to the stage of illness. The cost data were represented by means and standard deviation and the results were demonstrated by tabulation. All cost data are presented in Malaysian Ringgit (RM). The cost difference between early stage (Stage I) and late stage (Stage II–IV) was analysed using independent *t*-test.

**Results:**

The cost per patient increased with stage of CRC, from RM13,672 (USD4,410.30) for stage I, to RM27,972 (USD9,023.20) for Stage IV. The early stage had statistically significant lower cost compared to late stage *t*(2) = −4.729, *P* = 0.042. The highest fraction of the cost was related to surgery for Stage I, but was superseded by oncology day care treatment for Stages II–IV. CRC is a costly illness. From a provider perspective, the highest cost was found in Stages III and IV. The early stages conserved more resources than did the advanced stages of cancer.

**Conclusion:**

Early diagnosis and management of CRC, therefore, not only affects oncologic prognosis, but has implications for health care costs. This adds further justification to develop and implement CRC screening programmes in Malaysia.

## Introduction

Colorectal cancer (CRC) is one of the most common cancers worldwide (1). It is a disease of the elderly and males are more affected than females. As the third most diagnosed cancer globally (1.4 million, 9.4%), after lung cancer (1.8 million, 12.6%) and breast cancer (1.7 million, 10.5%), it is the fourth commonest cause of cancer deaths, accounting for 694,000 deaths in 2012 (8.5% of all deaths) (2).

The incidence of CRC is rising globally with time and its pattern differs in different income groups, where it commonly affects high income countries rather than low-income countries. In the USA, the incidence rate of CRC has been declined from 1998 to 2005, that decline was mainly attributed to the implementation of screening programme and the early detection of precancerous polyp (3).

In Peninsular Malaysia, the trends and the patterns of CRC varied in different times. According to the first National Cancer Registry Report (2003–2005), the CRC incidence has increased over the years (4). Among all types of cancer, colon cancer was the second highest, representing 11.9%, and it was the first high among males and the third among females, representing 14.5% and 9.9%, respectively (4). On the other hand, CRC was the second most diagnosed among both males and females in 2007 (5). The incidence was found higher among the Chinese population than among Malay and Indian ethnic groups (5).

Cancer burden worldwide resulted in treatment expenditures of USD217 billion in 2009. The cancers accounting for the highest aggregate cost for new cancer cases, and treatment expenditure globally, were: lung (USD86 billion), colorectal (USD39 billion), breast (USD25 billion), prostate (USD15 billion), and stomach (USD15 billion). While these cancers account for 85% of the global treatment expenditure, they are reported to comprise 55% of aggregate costs (6).

CRC, as well as its economic burden, is becoming a significant health problem in low-and middle-income countries, due to an increase in the incidence (7). This increase is due to the increase in the aging population and rapid changing in lifestyle as a result of economic development and epidemiological transition (8).

Knowing more about the economic aspect of CRC is important for building up evidence-based, economically feasible, and culturally proper preventive and treatment strategies to obtain better CRC health outcomes. Costing data for the management of each CRC Stage (I–IV) can show the possible cost differences between early and late stages, as the long-term prognosis of the CRC depends on the stage of the illness at the time of diagnosis.

Health care in Malaysia is mainly the responsibility of the Ministry of Health. Malaysia generally has an efficient and widespread system of health care that operates as a two-tier system consisting of a government-run universal health care system and a co-existing private health care system. The utilisation of public health services is highly subsidised for all Malaysian citizens, with only nominal charges being levied on certain services that the patients have to pay for out of their own pocket. Income from these charges (out of pocket-OOP) is one of the sources of health care funding (37 %). Other sources are taxes, Employee Provident Fund contributions, Social Security Organisation contributions and private insurance premiums (9).

In Malaysia there is limited data about the cost of CRC especially from the health care provider side. Therefore, this study aimed to find out the total first year cost of CRC management following diagnosis from a health care provider perspective at different Stages (I–IV).

## Methods

Data was collected at a tertiary hospital in Kuala-Lumpur, Malaysia, namely University of Malaya Medical Centre (UMMC) in a year 2012.

The study investigated the cost of illness of CRC management by using quantitative methods. The standard operating procedure (SOP) for CRC was developed for each stage according to national data and guidelines at the UMMC. While it was known that some patients might have individualised management, the developed SOP was projected to represent the treatment procedures of a ‘typical’ case at a particular stage. The SOP was reviewed and validated by the experts at UMMC (colorectal surgeons, oncologists).

**Figure 1:**
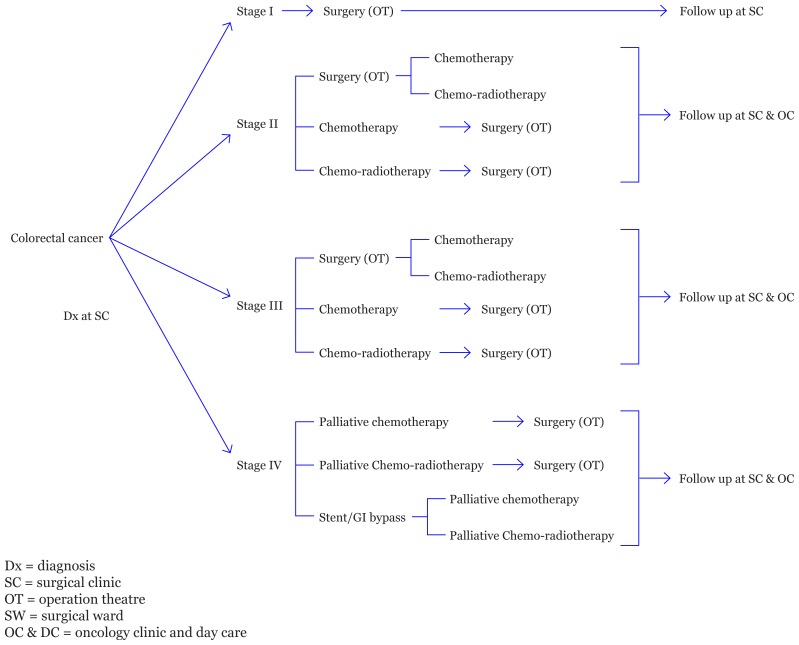
Standard operating procedure of colorectal cancer treatment at UMMC

Patients are usually referred to the surgical outpatient clinic (SC) for diagnosis and determination of operability. Following surgery, they are referred to the oncology clinic to decide on the appropriate chemotherapy or radiotherapy, which is often administered in day care. Following completion of treatment, patients are followed up with at the surgical clinic. Some patients are referred to the oncology department before the operation (Figure 1).

Very rarely, patients might develop post-chemotherapy side effects and may require admission to the oncology department. However, as we considered only typical cases and excluded the cost of complications, this area was not involved in my analysis.

To estimate the cost of resources used by the CRC patient, A combination of the top-down approach and activity-based costing was applied. The unit cost was calculated and incorporated into the treatment pathway in order to obtain the total cost of managing a single CRC patient according to the stage of illness.

The hospital cost was grouped into general overhead, specific overhead, consumables, equipment, medical supplies, support services, non-specific medical supplies, food, labour, investigation, radiological imaging costs, histopathology costs, and specific medication costs.

The general overhead cost includes the cost of services shared among all of the patients attending UMMC. The general overhead, in this study, included general equipment, non-medical staff salaries, administration and bought services costs, utility costs, cleaning and maintenance costs, depreciated assets costs and general supply costs (10). In order to get the general overhead cost per patient, the obtained total cost was divided by the total services used at UMMC-2012. However, the specific overhead cost is the cost of services at each specific area used by the CRC patients, namely the surgical clinic, surgical ward (7U), operation theatre, oncology clinic and day care. It may include utility cost, cleaning and maintenance costs, and the administration and bought services costs.

The cost of consumables was calculated for each area used by the CRC patients, taking into account the manufacturer’s wholesale price. It included the cost of gauze, syringe, gloves, tissues, plaster, etc.

The calculation of equipment costs began with the purchase price, which was then annualised according to its life span (11).

The hospital medication supply cost, nonspecific medical supply cost and support services per year were apportioned according to the type of care (outpatient or inpatient care). Afterwards, it was multiplied by the workload ratio of the each specific place used by the CRC patient. Finally, it was divided by the total number of clinic visits/patient days or procedures in the year 2012.

The cost of support services in UMMC included the cost of staff, utilities, administration/bought services, cleaning and maintenance and general supplies for each of laboratory unit, radiology department, pharmacy and rehabilitation unit. Nevertheless, nonspecific medical supply cost included the annual medical equipment supply cost.

In order to obtain the daily food cost per patient, the total food cost per year was divided by the total number of patient-days at the hospital. Then, the cost per patient-day was multiplied by the length of the CRC patient’s stay at the hospital. It was calculated only for the surgical ward. No meals were served to the patients at day care. The data were obtained from the dietetic department.

Labour cost was calculated by getting the total staff salary and divided it by the total number of patients in each specific place used for CRC treatment.

For the support clinic staff, consisting of sisters, nurses, attendants and clerks, the labour cost was calculated by obtaining the mean annual salary of each category. Then, it was multiplied by the total number of staff in that category. Finally, it was divided by the total number of clinic visits/patient days or procedures in the year 2012.

For the doctors, first the mean annual salary was calculated for both medical officers (MO) and housemen (HO), and then it was multiplied by 33%, which was the average time that was spent in the clinic, while same average duration of time was spent in each the operation theatre and the ward. Finally, the result was divided by the total number of clinic visits/patient days or procedures in the year 2012. For academic staff, the calculation was the same as for the doctors, but before being multiplied by 33%, first the total salary was multiplied by 60%, as supposedly they spend 60% of their total work time consulting with the patients and the remaining 40% is spent in teaching.

Specific tests performed for CRC patients, radiology images, histopathology examination and specific medication were obtained from the medical records of patients and approved by the experts (CRC surgeons and oncologists). Then, it was given to the laboratory unit, radiology department, histopathology unit pharmacy and financing department to obtain the cost of each. Then, the cost was multiplied by the frequency of doing that test to each patient during diagnosis and in the first year of treatment.

The cost data were represented by means and standard deviation and the results were demonstrated by tabulation. All the cost data are presented in Malaysian Ringgit (RM) and United States Dollar USD (USD1 = RM3.10, conversation rate of 2012) (12).

The study was approved at the Medical Ethic Committee of the University of Malaya Medical Centre, with the reference number 975.19 on 12 March, 2013.

## Health Care Utilisation

Complications can significantly escalate costs, but since serious complications are uncommon, we felt it would be more generalisable to prepare the SOP for uncomplicated cases.

Table 1 shows the average number of visits at the surgical and oncology clinics, the number of surgeries, and the number of admissions to each of surgical ward and day care per CRC patient per year.

## Statistical Analysis

The normality of cost data was checked using Shapiro-Wilk test. For normally distributed data, mean and standard deviation were used. However, for non-normally distributed data, median and interquartile range (IQR) were used. The independent *t*-test was utilised in order to find the cost difference between early stage (Stage I) and late stage (Stage II–IV). Our hypothesis was that there is a difference in a cost of management for CRC among four cancer stages.

## Result

The provider cost for CRC management consists of the cost of service provision at the outpatient surgical and the oncology clinic, running oncology day care, achieving surgical operations at the operation theatre and admission at the surgical ward (7U), at UMMC.

### Provider Cost at the Surgical Outpatient Clinic

The general overhead cost, specific overhead cost, consumables, equipment cost, medical supply, support service cost, nonspecific medical supply and labour costs were calculated by first obtaining the cost per one visit and then, in order to obtain the mean cost per CRC patient, multiplying it by the minimum (4) and maximum (6) number of CRC patient visits to the clinic per year (Table 1).

For investigation purposes, a specific test (the tumour marker, carcinoembryonic antigen (CEA) was assessed every three months during the first year of treatment and once during diagnosis, costing RM75 (15 × 5).

For radiology tests, the cost of computerised tomography (CT) scan was RM450 [USD145.20], and was recommended two to three times during the first year following the diagnosis in advanced cases (Stage IV). The cost of endoscopy is RM200 [USD64.50], which was recommended one time for diagnosis. The cost of biopsy examination was RM100 [USD32.30]. In all, the investigation cost for Stages I–III was [(RM450 + RM200 + RM100 = RM750 (USD241.90)]. For advanced cases (Stage IV), the minimum cost was [(2 × RM450) + (1 × RM200) + RM100] = RM1,200 (USD387.10)]. However, the maximum cost was [(3 × RM450) + (1 × RM200) + RM100] = RM1,650 (USD532.30)]. The mean radiological examination cost can be found in Table 2.

The general overhead, specific overhead, equipment, consumables, medical supply, support service, nonspecific medical supplies and staff cost were all expected to be identical for everybody as all patients at different stages were using the same facilities at the surgical clinic (Table 2).

For investigations, radiology images and histopathological examinations, the cost was calculated according to the range that those tests were requested per patient.

Table 2 shows the total mean cost per CRC treatment in the surgical clinic, which was RM1,580.30 [USD509.80] (min. RM1,428.40–max. RM1,731.20) for Stage I–III, and it was RM2,255.30 [USD727.40] (min. RM1,878.40–max. RM2,631.20) for Stage IV.

### Provider Cost at Oncology Outpatient Clinic

After multi-disciplinary clinical decision-making (CDM), the patient visits the oncology clinic to be informed of the appropriate treatment. Then, the patient visits the clinic before each chemotherapy or radiotherapy session to assess eligibility for treatment. The number of visits to the clinic is the same as the number of doses of chemotherapy—six for Stage II and 8–12 visits for each of Stage III and IV. Stage I patients were not referred to the oncology clinic.

The general overhead cost, specific overhead cost, consumables, equipment cost, medical supplies, support service cost, nonspecific medical supplies and labour cost were calculated by first obtaining the cost per one visit and then, in order to obtain the mean cost per CRC patient, multiplying it by six (number of visits) in Stage II and multiplying by the minimum (eight visits) and the maximum (12 visits) number of visits to the oncology clinic per year in each of Stage III and IV (Table 3).

In Stage II, the patient undergoes intravenous chemotherapy for five days every four weeks (Mayo’s regime) for a consecutive six months. In Stage III, the patient undergoes the Folfox or Xelox regimen. Before starting chemotherapy, the patient is requested to do the Carcinoembryonic Antigen (CEA) tumour marker, which costs RM15. With that, the total investigation cost for Stages II and III is RM15 [USD4.80]. For Stage IV, the patient is requested to do CEA once before starting chemotherapy and then every two cycles for monitoring. Therefore, if the patient undergoes the Folfox regimen, which consists of 12 sessions, the investigation tests cost RM165 (15 × 7) [USD53.20]. Likewise, if the patient undergoes the Xelox regimen, which consists of eight sessions, the investigation will cost RM115 (15 × 5) [USD37.10]. The CT scan is requested once after three or four chemotherapy sessions only for Stage IV patients. For that, it was requested three to four times depending on the type of chemotherapy regime (Table 3).

Table 3 shows the mean total cost according to stage. There was a rise in mean costs, which is demonstrated as the stage increases from II to IV.

### Provider Cost at the Oncology Day Care

The cost of day care varied according to the prescribed treatment, either chemotherapy or chemo-radiotherapy treatment, depending on the different stages and sites of CRC.

For CRC patients undergoing chemotherapy, the cost of general overhead, specific overhead, consumables, equipment, medical supplies, non-specific medical supplies and labour per visit was calculated according to range number of visits. The medication cost was already calculated per regime. The medication cost of Mayo’s regimen was RM679.20 [USD219.10]. However, for Stages III and IV, there are two common regimes used (Xelox or Folfox), and the medication cost was the mean cost of the two regimens (RM4,174.10, RM1,0214.40), and was found to be RM7,194.20 (SD: 4,271.10) (Table 4).

The mean total cost of chemotherapy treatment at day care for Stage III and IV was RM9,129.20 [USD2,944.90] (SD: 3,723.80, min. RM6,496–max. RM11,762.30). For CRC patients undergoing chemo-radiotherapy, the values of cost components were multiplied by 25; which was the number of patient visits in all of the cost components except for the labour and medication cost. The labour cost was RM2,352.70 [USD758.90] for simulation, which was done once for all patients. For radiotherapy, the labour cost was RM125.90 [USD 40.60] per one session, which was multiplied by the number of sessions, 25 (RM125.90 × 25 = RM3,147.50 [USD1,015.30]). In most cases, radiotherapy is either associated with intravenous chemotherapy or with oral chemotherapy. Few cases are only given short course radiotherapy alone; those cases are not included in the calculation. The cost in either case varied. For associated intravenous chemotherapy the patient undergoes 10 sessions, and the labour cost of associated chemotherapy treatment was (RM44.40 × 10 = RM444) [USD143.20] (Table 5).

Table 5 and Table 6 demonstrate the two costs for treating CRC patients with chemoradiotherapy at UMMC. This is because there are two approaches involved—once is radiotherapy with intravenous chemotherapy and the other approach is radiotherapy with oral chemotherapy (Capecitabine). The two costs were different as the expense of chemotherapy, labour, and the numbers of hospital visits differ.

So from the above tables, the cost of chemoradiotherapy treatment at day care ranged from RM9,778.50, for the radiotherapy with intravenous chemotherapy, and RM11,233.30 for radiotherapy with oral chemotherapy management. The mean cost of chemoradiotherapy treatment at day care was RM10,505.90 [USD3,389] (SD: 1,028.70). On the other hand, the mean cost of treating CRC patients at day care with either chemotherapy or with both chemo-radiotherapy was the average between the mean cost of chemotherapy and that of chemo-radiotherapy, which was RM8,494.90 [USD2,740.30] (SD: 2,844) for Stage II and RM9,817.60 [USD3,167] (SD: 973.50) for each of Stage III and IV.

### Provider Cost at a Surgical Ward (7U)

The cost at the surgical ward was calculated according to the minimum and the maximum length of stay at the ward for a CRC patient, which was three to ten day stay according to expert opinion. All of the cost components that were obtained per patient day were multiplied by the minimum and the maximum lengths of stay for a CRC patient to get the mean cost per CRC patient at the ward (Table 7).

Table 7 shows the CRC treatment cost per admission. Because the CRC patient has a range of one to three admissions per year, the final total cost at the surgical ward was the mean cost between the minimum cost (1 × RM4,052.90) and the maximum cost (3 × RM4,052.90), which was RM8,105.80 [USD2,614.80](SD: 5,731.70).

### Provider Cost at the Operation Theatre

The cost of the operation theatre was calculated according to the minimum and maximum number of surgeries done for a CRC patient.

The general overhead was already calculated for the surgical ward; therefore it was not included again among the operation theatre cost components. For all others cost components, the cost per procedure was multiplied by the minimum and maximum number of surgeries (one to three) done for a CRC patient.

The labour cost was the sum of the support staff cost and the colorectal surgeon staff per procedure is RM354.20 [USD114.30]. Then in order to get the mean cost, it was multiplied by the minimum and maximum number of surgeries (one to three) done for a CRC patient (Table 8).

The total mean cost at the operation theatre was RM3,800.30 [USD1,225.9] (min. RM2,152.40–max. RM5,448.20).

### Annual Provider Cost Per Colorectal Cancer Patient

The total provider cost per CRC patient varied according to stage of cancer. Costs increased with stage of CRC, from RM13,672 (USD4,410.30) for Stage I, to RM27,972 (USD9,023.20) for Stage IV (Table 9 & Table 10).

The early stage had statistically significant lower cost compared to late stage *t*(2)= −4.729, *P* = 0.042. The highest fraction of the cost was related to surgery for Stage I, but this was superseded by oncology day care treatment for Stages II–IV (Table 10).

## Discussion

At present, there are no published data reporting health care provider cost of CRC management in Malaysia and other Asian or middle-income countries. I have demonstrated that cost increases with increasing stage, consistent with data from developed European countries (13).

However, surgery-related costs do not seem to be affected by increasing stage. This may be because the surgical procedures, post-operative care and length of hospital stay are similar regardless of stage. On the other hand, the exclusion of cost of complications may have resulted in a failure to capture rising costs of more complex surgeries, which tend to be associated with more advanced tumours and a higher rate of complications.

Stage I patients are not referred to oncology departments, so the stage I cost for the oncology clinic and day care was found to be zero, which was the same reported in a study done in Ireland (14). In contrast to Stages II–IV, of which the cost varied based on the prescribed chemotherapy regime or of chemotherapy and radiotherapy together.

In the oncology day care, the cost of medication was that of the conventional chemotherapy, while most of the newly published papers included the cost of monoclonal antibodies as well. Monoclonal antibodies have been accepted for the management of advanced CRC in several developed countries in Asia, such as Singapore, Korea and Japan (15). In Malaysia, however, even though it is listed with the National Pharmaceutical Control Bureau Ministry of Health, until now, it has not been registered in the Ministry of Health Drug Formulary because of the insufficient evidence of its usefulness, in addition to cost effectiveness, within the country. As a result, it cannot be used in public hospitals.

Highest percentage of provider cost was due to inpatient stay and that was consistent with some studies done worldwide (13, 16).

There was an underestimation of the cost from the provider perspectives, because the building cost of the hospital (UMMC) was not included in this cost analysis due to unavailability of the data since UMMC is an old hospital and was built in 1967, making it difficult to trace the cost.

### International Comparison

Over the last 15 years, a number of studies have collected stage specific costs of CRC management in US, France and study compare between five countries (Belgium, France, Germany, Italy and the UK) (13, 17–19). In addition to these, a review of studies conducted over a 10 year period of time (1996–2006) in North America and Europe, found that it is hard to compare the costs among studies. That is because of the variation in cost perspective, the variety of costs involved, the duration of follow-up, disease recurrence and/or surveillance that was incorporated. Moreover, the different diagnostic and treatment pathways have changed over time (20).

### Limitation

Assessing resource utilisation precisely is a challenge for every cost-of-illness study. Perfectly, data should be collected individually and prospectively from a representative group of cases. However, this is not always possible. Commonly, researchers have to make a balance between gathering very comprehensive data on a low numbers of subjects and using less comprehensive data on a great numbers of cases.

In this study, the calculation was based on making a number of assumptions, in order to come up with the SOP and a mean cost.

As the study was limited to the cost of the first year following diagnosis, a lifelong cost study could be more valuable, as it could calculate the cost of recurrence, as well as the survival rate of the respondents. In spite of that, the main components of the cost (direct and indirect) were calculated, and we considered the most costly phase of the treatment, which was the first year following diagnosis (19, 21).

Economic studies can be achieved using a number of different ways and approaches. A review of cost studies revealed that there is a lack of available and published standards for performing and reporting cost analysis (22).

Determining the approach depends on the availability of data. In this study, a mixed costing methodology (top-down and activity-based approaches) was used to calculate the provider cost. A mixed methodology can be a possible alternative to standard top–down, and bottom-up approaches and is frequently used in lower-and middle-income country (LMICs) settings due to the unavailability of data (23). The flexibility of a mixed costing methodology permits using all accessible data sources and tolerates making balances between more accurate micro-costing and more feasible methodologies to measure cost items (24). This leads to comprehensive cost estimates for a specific disease (24). It is reliable because all important cost components are recognised and valued at a proper detailed level. This allows for the detection of costs per patient and for involving sub-populations that might have the greatest share in the total costs. In addition, this study focus mainly on health care cost of CRC management, for that it is difficult of give a suggestion related to administration.

### Application of Study Findings

The results of this study provide information on the amount that can be saved by early diagnosis and management of CRC, which affects the prognosis of CRC patients, depending on the stage at the time of diagnosis.

This result is essential from the public health aspect because it gives a strong justification for the implementation of colorectal screening programmes and other interventions. The target of screening is to have a positive effect on a shift in the stage at the time of diagnosis, from late to early stages. Internationally, especially in developed countries, after the establishment of colorectal organised screening programme there is changing a trend of diagnosis from late to early and improvement of survivorship (25, 26). Policymakers need cost estimated data to rationally allocate health care resources in time to improve and expand the accessibility of the services.

## Conclusion

CRC is a costly illness. From a provider perspective, the highest cost was found in stages III and IV. The early stages conserved more resources than did the advanced stages of cancer. Highest costs were found due to inpatient stays at the surgical ward for each of Stage I and II and due to oncology day care admission in each of Stage III and IV. The lowest cost was found due to outpatient visits.

Early diagnosis and management of CRC, therefore, not only affects oncologic prognosis, but has implications for health care costs. This adds further justification to develop and implement CRC screening programmes in Malaysia.

## Figures and Tables

**Table 1:** Number of utilisation of different health care services of CRC patients at UMMC

Area	Service utilisation (median, range)			

	Stage I	Stage II	Stage III	Stage IV
• Surgical clinic	5 (4–6)	5 (4–6)	5 (4–6)	5 (4–6)
• Oncology clinic	0	6	10 (8–12)	10 (8–12)
• Oncology day care	0	30	10 (8–12)	10 (8–12)
• Surgical ward (7U)				
i) Number of admission	2 (1–3)	2 (1–3)	2 (1–3)	2 (1–3)
ii) Number of days/admission	6.5 (3–10)	6.5 (3–10)	6.5 (3–10)	6.5 (3–10)
• Operation theater	2 (1–3)	2 (1–3)	2 (1–3)	2 (1–3)

**Table 2:** Total provider cost for treating CRC patient at surgical clinic at different stages, UMMC-2012 (Ringgit Malaysia)

Mean (SD)				

Cost components	Stage I cost	Stage II cost	Stage III cost	Stage IV cost
General overheads	275.50 (77.9)	275.50 (77.9)	275.50 (77.9)	275.50 (77.9)
Specific overheads	14.00 (4)	14.00 (4)	14.00 (4)	14.00 (4)
Consumables	10.50 (2.9)	10.50 (2.9)	10.50 (2.9)	10.50 (2.9)
Equipment	4.70 (1.3)	4.70 (1.3)	4.70 (1.3)	4.70 (1.3)
Medical supply	117.50 (33.2)	117.50 (33.2)	117.50 (33.2)	117.50 (33.2)
Support services	101.50 (28.7)	101.50 (28.7)	101.50 (28.7)	101.50 (28.7)
Unspecific medical supply	24.00 (6.7)	24.00 (6.70)	24.00 (6.7)	24.00 (6.7)
Labour	207.50 (58.6)	207.50 (58.6)	207.50 (58.6)	207.50 (58.6)
Investigations	75.00	75.00	75.00	75.00
Radiological images	750.00	750.00	750.00	1,425.00 (318.2)

**Total cost per CRC patient**	**1,580.00 (750.2)**	**1,580.00 (750.2)**	**1,580.00 (750.2)**	**2,255.30 (1020.6)**

**Table 3:** Total provider cost for treating CRC patient at oncology clinic at different stages, UMMC-2012 (Ringgit Malaysia)

Cost components	Mean (SD)			

	Stage I cost	Stage II cost	Stage III cost	Stage IV cost
General overheads	NA*	330.50	550.90 (155.8)	550.90 (155.8)
Specific overheads	NA	23.40	39.00 (11.0)	39.00 (11.0)
Consumables	NA	109.30	183.00 (51.7)	183.00 (51.7)
Equipment	NA	261.00	435.00 (123.0)	435.00 (123.0)
Medical supply	NA	141.00	235.00 (66.5)	235.00 (66.5)
Support service cost	NA	121.80	203.00 (57.4)	203.00 (57.4)
Unspecific medical supply	NA	28.80	48.00 (13.6)	48.00 (13.6)
Labour	NA			
• For CMD	NA	76.60	76.60	76.60
• For clinic visits	NA	414.60	691.00 (195.4)	691.00 (195.4)
Investigations	NA	15.00	15.00	140.00 (35.3)
Radiological images	NA	0	0	1575.00 (318.2)

Total cost/patient	NA	1,522.00 (8,99.3)	2,303.50 (2,012.2)	3,993.50 (3,109.4)

* NA: not applicable

**Table 4:** Provider cost for treating CRC patient with chemotherapy at oncology day care at different stages, UMMC-2012 (Ringgit Malaysia)

Cost components	Mean (SD)			

	Stage I cost	Stage II cost	Stage III cost	Stage IV cost
General overheads	NA*	1,652.70	550.90 (155.8)	550.90 (155.8)
Specific overheads	NA	117.00	39.00 (11.0)	39.00 (11.0)
Consumable	NA	549.00	183.00 (51.1)	183.00 (51.1)
Equipment	NA	1305.00	435.00 (123.0)	435.00 (123.0)
Medical supply	NA	705.00	235.00 (66.5)	235.00 (66.5)
Unspecific medical supply	NA	144.00	48.00 (13.6)	48.00 (13.6)
Labour	NA	1,332.00	444.00 (125.6)	444.00 (125.6)
Medication	NA	679.20	7,194.20 (4271.1)	7,194.20 (4271.1)

Total cost per CRC patient	NA	6,483.90 (4,983.3)	9,129.20 (8,880.0)	9,129.20 (8,880.0)

* NA: not applicable

**Table 5:** Provider cost for treating CRC patient at oncology day care with radiotherapy and intravenous chemotherapy at different stages, UMMC-2012 (Ringgit Malaysia)

Cost components	Mean (SD)			

	Stage I cost	Stage II cost	Stage III cost	Stage IV cost
General overheads	NA*	1,377.30	1,377.30	1,377.30
Specific overheads	NA	97.50	97.50	97.50
Consumable	NA	457.50	457.50	457.50
Equipment	NA	1,087.50	1,087.50	1,087.50
Medical supply	NA	587.50	587.50	587.50
Unspecific medical supply	NA	120.00	120.00	120.00
Labour	NA			
• For simulation	NA	2,325.70	2,325.70	2,325.70
• For radiotherapy	NA	3,147.50	3,147.50	3,147.50
• For IV chemotherapy	NA	444.00	444.00	444.00
Medication	NA	134.00	134.00	134.00

**Total cost per patient**	NA	9,778.50 (7,201.7)	9,778.50 (7,201.7)	9,778.50 (7,201.7)

* NA: not applicable

**Table 6:** Provider cost for treating CRC patient at oncology day care with radiotherapy and oral chemotherapy at different stages, UMMC-2012 (Ringgit Malaysia)

Cost components	Mean (SD)			

	Stage I cost	Stage II cost	Stage III cost	Stage IV cost
General overheads	NA*	1,377.30	1,377.30	1,377.30
Specific overheads	NA	97.50	97.50	97.50
Consumable	NA	457.50	457.50	457.50
Equipment	NA	1,087.50	1,087.50	1,087.50
Medical supply	NA	587.50	587.50	587.50
Unspecific medical supply	NA	120.00	120.00	120.00
Labour				
• For simulation	NA	2,325.70	2,325.70	2,325.70
• For radiotherapy	NA	3,147.50	3,147.50	3,147.50
• For oral chemotherapy	NA	0	0	0
Medication	NA	2,032.80	2,032.80	2,032.80

**Total cost per CRC patient**	NA	11,233.30 (1,0519.2)	11,233.30 (1,0519.2)	11,233.30 (1,0519.2)

* NA: not applicable

**Table 7:** Provider cost for treating CRC patient at surgical ward at different stages, UMMC-2012 (Ringgit Malaysia)

Cost components	Mean (SD)			

	Stage I cost	Stage II cost	Stage III cost	Stage IV cost
General overheads	358.10 (272.7)	358.10 (272.7)	358.10 (272.7)	358.10 (272.7)
Specific overheads	65.70 (50.0)	65.70 (50.0)	65.70 (50.0)	65.70 (50.0)
Consumable	899.60 (685)	899.60 (685)	899.60 (685)	899.60 (685)
Equipment	20.20 (15.3)	20.20 (15.3)	20.20 (15.3)	20.20 (15.3)
Medical supply	755.30 (575.2)	755.30 (575.2)	755.30 (575.2)	755.30 (575.2)
Support services	653.90 (497.9)	653.90 (497.9)	653.90 (497.9)	653.90 (497.9)
Unspecific medical supply	154.10 (117.3)	154.10 (117.3)	154.10 (117.3)	154.10 (117.3)
Food	158.00 (120.3)	158.00 (120.3)	158.00 (120.3)	158.00 (120.3)
Labour	988.00 (752.4)	988.00 (752.4)	988.00 (752.4)	988.00 (752.4)

**Total cost/patient**	4,052.90 (3,305.1)	4,052.90 (3,305.1)	4,052.90 (3,305.1)	4,052.90 (3,305.1)

**Table 8:** Provider cost for treating CRC patient at operation theatre at different stages, UMMC-2012 (Ringgit Malaysia)

Cost components	Mean (SD)			

	Stage I cost	Stage II cost	Stage III cost	Stage IV cost
Specific overheads	26.80 (18.9)	26.80 (18.9)	26.80 (18.9)	26.80 (18.9)
Consumables	618.20 (437.1)	618.20 (437.1)	618.20 (437.1)	618.20 (437.1)
OT supply	687.00 (485.8)	687.00 (485.8)	687.00 (485.8)	687.00 (485.8)
Medical supply	54.00 (38.2)	54.00 (38.2)	54.00 (38.2)	54.00 (38.2)
Unspecific medical supply	11.00 (7.8)	11.00 (7.8)	11.00 (7.8)	11.00 (7.8)
Sterile services	8.60 (6.1)	8.60 (6.1)	8.60 (6.1)	8.60 (6.1)
Labour	708.40 (500.9)	708.40 (500.9)	708.40 (500.9)	708.40 (500.9)
Histopathology test	500.00	500.00	500.00	500.00
Medication	1,186.30 (835.7)	1,186.30 (835.7)	1,186.30 (835.7)	1,186.30 (835.7)

**Total cost/surgery**	3,800.30 (2,643.6)	3,800.30 (2,643.6)	3,800.30 (2,643.6)	3,800.30 (2,643.6)

**Table 9:** Total annual provider cost at different stages of CRC at UMMC-2012 (min., max.) (Ringgit Malaysia)

CRC stage	Cost (min., max.)					Total

	Surgical clinic	Oncology clinic	Oncology day-care	Surgical ward	Operation theatre	
Stage I	1,428.40	NA	NA	4,052.90	2,152.40	7,633.70
	1,731.20	NA	NA	12,158.70	5,448.20	19,338.10
Stage II	1,428.40	1,522.00	6,483.90	4,052.90	2,152.40	15,639.60
	1,731.20	1,522.00	10,505.90	12,158.70	5,448.20	31,366.00
Stage III	1,428.40	1,686.40	9,129.20	4,052.90	2,152.40	18,449.30
	1,731.20	2,920.60	10,505.90	12,158.70	5,448.20	32,764.60
Stage IV	1,878.40	3,126.40	9,129.20	4,052.90	2,152.40	20,339.30
	2,631.20	4,860.60	10,505.90	12,158.70	5,448.20	35,604.70

**Table 10:** Total annual provider cost per CRC patient at UMMC-2012 (Ringgit Malaysia)

Place	Mean cost (RM) (%)			

	Stage I	Stage II	Stage III	Stage IV
Surgical clinic	1,580.00 (11.7%)	1,580.00 (6.7%)	1,580.00 (6.2 %)	2,254.80 (8.1%)
Oncology clinic	0	1,522.00 (6.5 %)	2,303.50 (9.0 %)	3,978.50 (14.2%)
Oncology day care	0	8,494.90 (36.1%)	9,817.60 (38.4 %)	9,817.60 (35.1%)
Surgical ward	8,105.80 (60.1%)	8,105.80 (34.5%)	8,105.80 (31.6 %)	8,105.80 (29%)
Operation theatre	3,800.30 (28.2%)	3,800.30 (16.2%)	3,800.30 (14.8%)	3,800.30 (13.6%)
Total cost per CRC patient (mean, SD)	13,485.90 (8,276.3)	23,502.80 (11,120.2)	25,606.90 (10,122.4)	27,972.00 (10,794.3)
